# *Nyssorhynchus dunhami*: bionomics and natural infection by *Plasmodium falciparum* and *P. vivax* in the Peruvian Amazon

**DOI:** 10.1590/0074-02760180380

**Published:** 2018-12-03

**Authors:** Catharine Prussing, Sara A Bickersmith, Marta Moreno, Marlon P Saavedra, Freddy Alava, Maria Anice Mureb Sallum, Dionicia Gamboa, Joseph M Vinetz, Jan E Conn

**Affiliations:** 1 University at Albany, State University of New York, School of Public Health, Department of Biomedical Sciences, Albany, NY, USA; 2 Wadsworth Center, New York State Department of Health, Albany, NY, USA; 3 University of California San Diego, Department of Medicine, Division of Infectious Diseases, La Jolla, CA, USA; 4 Universidad Peruana Cayetano Heredia, Facultad de Ciencias y Filosofia, Laboratorios de Investigación y Desarrollo, Laboratorio ICEMR-Amazonia, Lima, Peru; 5 Ministry of Health, Iquitos, Peru; 6 Universidade de São Paulo, Faculdade de Saúde Pública, Departamento de Epidemiologia, São Paulo, SP, Brasil; 7 Universidad Peruana Cayetano Heredia, Instituto de Medicina Tropical Alexander von Humboldt, Lima, Peru

**Keywords:** *Nyssorhynchus nuneztovari* s.l., Nyssorhynchus dunhami, Plasmodium, larval habitats, Peruvian Amazon

## Abstract

BACKGROUND *Nyssorhynchus dunhami*, a member of the Nuneztovari Complex, has been collected in Brazil, Colombia, and Peru and described as zoophilic. Although to date *Ny. dunhami* has not been documented to be naturally infected by *Plasmodium*, it is frequently misidentified as other Oswaldoi subgroup species that are local or regional malaria vectors. OBJECTIVES The current study seeks to verify the morphological identification of Nuneztovari Complex species collected in the peri-Iquitos region of Amazonian Peru, to determine their *Plasmodium* infection status, and to describe ecological characteristics of their larval habitats. METHODS We collected *Ny. nuneztovari* s.l. adults in 2011-2012, and *Ny. nuneztovari* s.l. larvae and adults in 2016-2017. When possible, samples were identified molecularly using cytochrome c oxidase subunit I (*COI*) barcode sequencing. Adult *Ny. nuneztovari* s.l. from 2011-2012 were tested for *Plasmodium* using real-time PCR. Environmental characteristics associated with *Ny. nuneztovari* s.l. larvae-positive water bodies were evaluated. FINDINGS We collected 590 *Ny. nuneztovari* s.l. adults and 116 larvae from eight villages in peri-Iquitos. Of these, 191 adults and 111 larvae were identified by *COI* sequencing; all were *Ny. dunhami*. Three *Ny. dunhami* were infected with *P. falciparum*, and one with *P. vivax*, all collected from one village on one night. *Ny. dunhami* larvae were collected from natural and artificial water bodies, and their presence was positively associated with other Anophelinae larvae and amphibians, and negatively associated with people living within 250m. MAIN CONCLUSIONS Of Nuneztovari Complex species, we identified only *Ny. dunhami* across multiple years in eight peri-Iquitos localities. This study is, to our knowledge, the first report of natural infection of molecularly identified *Ny. dunhami* with *Plasmodium.* We advocate the use of molecular identification methods in this region to monitor *Ny. dunhami* and other putative secondary malaria vectors to more precisely evaluate their importance in malaria transmission.

*Nyssorhynchus nuneztovari* s.l. (formerly named *Anopheles nuneztovari* s.l.)^1^ is a species complex consisting of at least four species: *Ny. nuneztovari* s.s. Gabaldón, 1940 (previously *Ny. nuneztovari* cytotypes B and C), which has been found in Colombia and western Venezuela; *Ny. nuneztovari* cytotype A, from the western Brazilian Amazon (Sant’Ana et al., manuscript in preparation); *Nyssorynchus goeldii* Rozeboom & Gabaldón, 1941, a genetically diverse species distributed throughout the Brazilian Amazon; and *Nyssorynchus dunhami* Causey, 1945, recorded from the Brazilian, Colombian, and Peruvian Amazon.^1^ ^,^ ^2^ ^,^ ^3^ Members of this complex have been incriminated in malaria transmission in their endemic areas, though documentation of their vector status has been complicated by their morphological similarities and changes in taxonomic status.^2^ ^,^ ^4^ ^,^ ^5^ *Ny. nuneztovari* s.s. is a major malaria vector in Colombia^6^ ^,^ ^7^ and Venezuela.^8^ ^,^ ^9^ ^,^ ^10^ Specimens of *Ny. nuneztovari* s.l. have been found to be naturally infected with *Plasmodium vivax* and *P. falciparum* in the Brazilian Amazon and French Guiana, but documentation of the extent of their involvement in malaria transmission in these areas is fairly restricted.^11^ ^,^ ^12^ ^,^ ^13^ ^,^ ^14^

*Ny. dunhami* was first described in 1945, based on specimens collected in Tefé, Amazonas, Brazil.^15^ This species is morphologically similar to other species in the Nuneztovari Complex, and has been considered synonymous with, and subsequently resurrected from synonymy with, both *Ny. nuneztovari* ^16^ and *Nyssorynchus trinkae* Faran, 1979.^17^ It has been collected from Amazonas,^2^ ^,^ ^5^ ^,^ ^11^ ^,^ ^15^ ^,^ ^17^ ^,^ ^18^ Acre,^19^ Pará,^18^ and Rondônia^18^ states, Brazil; Amazonas department, Colombia;^20^ and Loreto department, Peru^21^ [Fig. 1; Supplementary data (Table I)]. *Ny. dunhami* is thought to be primarily zoophilic and has not been incriminated as a malaria vector.^2^ ^,^ ^22^ However, the geographic distributions of *Ny. dunhami*, *Ny. goeldii*, and *Ny. nuneztovari* A overlap,^18^ and the reliance on morphological identification in previous reports of *Plasmodium* infection in *Ny. nuneztovari* s.l.^23^ ^,^ ^24^ could confound our current understanding of the vector status of *Ny. dunhami*.

In Peru, *Ny. nuneztovari* s.l. specimens have been reported from the departments of Pasco, Junín, Loreto, Ucayali, and Madre de Dios.^25^ ^,^ ^26^ ^,^ ^27^ ^,^ ^28^ In addition, *Ny. trinkae*, which was previously considered synonymous with *Ny. dunhami*,^17^ was identified morphologically and found infected with *Plasmodium* in the Junín^29^ and Madre de Dios departments.^25^ In 2015, *Ny. dunhami* was first reported from the peri-Iquitos area of Loreto, Peru,^21^ where malaria is endemic and seasonal, and the primary vector is *Ny. darlingi* Root, 1926.^21^ ^,^ ^28^ ^,^ ^30^. Despite the predominance of *Ny. darlingi* in several Loreto department communities, other Anophelinae species, including *Ny. nuneztovari* s.l. species, might be local or seasonal malaria vectors in this region. To investigate the vector status of the Nuneztovari Complex in Loreto, and inform future vector control efforts, the aims of the current study were to identify *Ny. nuneztovari* s.l. species in the peri-Iquitos area, to determine their *Plasmodium* infection status, and to characterize their larval ecology.

## MATERIALS AND METHODS

*Adult mosquito collections: 2011-2012* - Adult *Ny. nuneztovari* s.l. mosquitoes were identified in the community of San José de Lupuna (LUP), as described previously.^21^ Adult human landing catch (HLC) collections were conducted inside and outside human dwellings (for the purposes of this paper, we define outside as in the peridomestic environment, within 10 m of a house), Shannon trap collections were conducted outside, and CDC light trap collections were conducted inside over 48 nights in LUP from 2011-2012. Details of collection methods are shown in Supplementary data (Table II). All mosquitoes were identified morphologically,^31^ ^,^ ^32^ ^,^ ^33^ then stored dry on silica gel until DNA extraction.

**Fig. 1:**
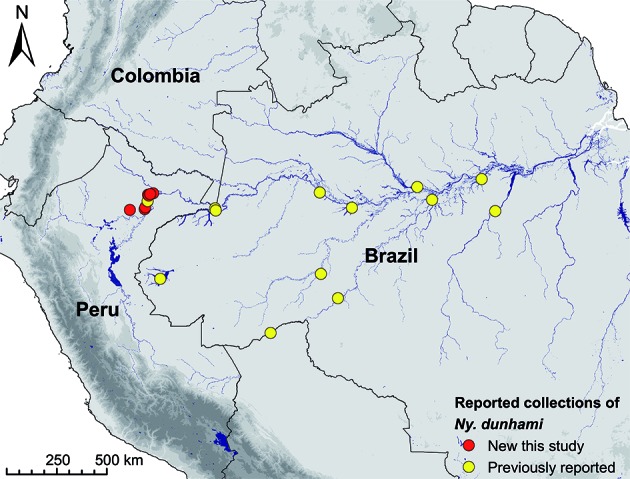
all recorded collection locations of *Nyssorhynchus dunhami*, including the current study. Coordinates and references are listed in Supplementary data (Table I).

**Fig. 2:**
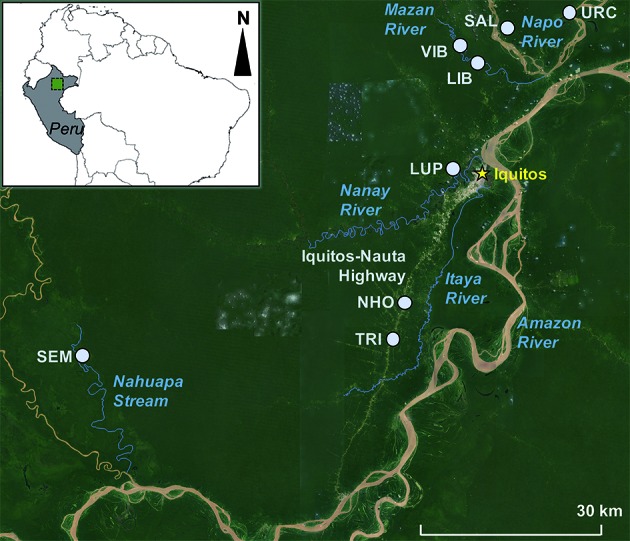
collection sites of adult and larval *Nyssorhynchus nuneztovari* s.l. in peri-Iquitos, 2011-2012 and 2016-2017. LUP: Lupuna; NHO: Nuevo Horizonte; TRI: El Triunfo; SEM: Santa Emilia; SAL: Salvador; URC: Urco Miraño; LIB: Libertad; VIB: Visto Bueno.

*Larval and adult mosquito collections: 2016-2017* - Collections of Anophelinae larvae and adults were conducted in eight villages in 2016-2017: LUP on the Nanay River, Nuevo Horizonte (NHO) and El Triunfo (TRI) on the Iquitos-Nauta Highway, Santa Emilia (SEM) on the Nahuapa Stream, Salvador (SAL) and Urco Miraño (URC) on the Napo River, and Libertad (LIB) and Visto Bueno (VIB) on the Mazan River [Fig. 2; coordinates in Supplementary data (Table I)]. In LUP, NHO, TRI, and SEM, six collections were conducted, and in SAL, URC, LIB, and VIB, five collections were conducted (Table I).

All potential larval habitats within a 1 km radius of each village were identified on satellite images and by ground-truthing, for a total of 88 water bodies in the eight villages. The 1 km distance was chosen to correspond approximately with the flight range of *Ny. darlingi*, the primary target of the larval collections.^34^ Each water body was sampled for Anophelinae larvae once per collection. Dips with standard 350 mL capacity dippers were taken 10 meters apart along the perimeter of each water body, with at most 20 dipping locations per site. At each dipping location, 10 dips were taken and examined for the presence of Anophelinae larvae.

Larvae from LUP, NHO, TRI, and SEM were reared to adults for morphological species identification. All larvae that died before reaching adulthood, that were unable to be identified, or that were identified as non-*Ny. darlingi*, were preserved for DNA extraction and molecular identification in 100% ethanol (larvae) or on silica gel (adults). All larvae from SAL, URC, LIB, and VIB were killed and preserved in 100% ethanol for molecular identification.

**TABLE I:** Dates of 2016-17 larval and adult collections in eight villages in peri-Iquitos, Peru

Collection	LUP, NHO, TRI, and SEM	SAL, URC, LIB, and VIB
1	January/February 2016 (rainy season)	March 2016 (rainy season)
2	April 2016 (rainy season)	June 2016 (rainy season)
3	July/August 2016 (dry season)	September 2016 (dry season)
4	September/October 2016 (dry season)	November 2016 (dry season)
5	November/December 2016 (dry season)	February/March 2017 (rainy season)
6	January-March 2017 (rainy season)	

LUP: Lupuna; NHO: Nuevo Horizonte; TRI: El Triunfo; SEM: Santa Emilia; SAL: Salvador; URC: Urco Miraño; LIB: Libertad; VIB: Visto Bueno.

Water body characteristics, including water body type, depth, cloud cover, light intensity (using a Foot Candle/Lux meter, Extech, Nashua, NH, USA), shade level, presence of vegetation, shade, presence of fish and amphibians, and type of water bed material, were recorded at each water body at each collection. In addition, chemical characteristics, including alkalinity, hardness, and nitrate and nitrite levels (using Eco-Check 5-in-1 Test Strips, Industrial Test Systems, Inc., Rock Hill, SC, USA); and pH, temperature, conductivity, and salinity (using an ExStik pH/Conductivity Meter, Extech, Nashua, NH, USA), were measured for each water body at each collection.

Concurrent with larval collections in SAL, URC, LIB, and VIB, adult mosquitoes were collected by HLC (12-hour collections inside and outside from 6 pm-6 am, two nights per month) and barrier screens (12-hour collections from 6 pm-6 am, one night per month) (Saavedra et al., manuscript in preparation). All adult mosquitoes collected that were initially morphologically identified as non-*Ny. darlingi* were molecularly identified.

*DNA extraction and molecular identification* - Total genomic DNA was extracted from the head/thorax of adult female mosquitoes, and from whole mosquito larvae and whole adults reared from larvae, using the DNeasy Blood & Tissue kit (Qiagen, Hilden, Germany). Mosquitoes were identified using polymerase chain reaction-restriction fragment length polymorphism (PCR-RFLP) of the ribosomal internal transcribed spacer 2 (ITS2) region,^26^ or by sequencing the barcode region of the cytochrome c oxidase subunit I (*COI*) gene.^35^ ^,^ ^36^ For each sample, the barcode region was sequenced in one direction using the forward primer by the Wadsworth Center Applied Genomic Technologies Core (New York State Department of Health). The sequences were queried against the BOLD Identification System^37^ or GenBank (https://www.ncbi.nlm.nih.gov/genbank/).

*Testing for Plasmodium infection* - All heads/thoraces of adult mosquitoes collected in 2011-2012 and molecularly identified as *Ny. nuneztovari* s.l. were tested for *Plasmodium* infection by real-time PCR of the small subunit of the 18S rRNA gene, as described previously.^38^ All mosquitoes were tested individually for *P. falciparum* and *P. vivax* using a triplex TaqMan assay (Life Technologies, Carlsbad, CA, USA).

*Larval site characteristics statistical analysis* - Analysis of the characteristics associated with the presence of *Ny. nuneztovari* s.l. larvae in water bodies was conducted in R v. 3.5.0^39^ at the level of the water body with a dataset that included one entry for each water body at each time point. Entries for water bodies that were dry at the time of collection were excluded from the final analysis. Water bodies that were dry at one or more collections were considered temporal, otherwise, they were considered permanent. Potential variables included locality, quarter (January-March 2016, April-June 2016, etc.), water body type (active fishpond vs. abandoned fishpond or natural site); presence of trees, bushes, grass, algae, emergent vegetation, floating vegetation, fish, and amphibians; presence of non-*Ny. nuneztovari* s.l. *Nyssorhynchus*, *Stethomyia*, or *Anopheles* spp. larvae; shade level (total, partial, or none); cloud cover (cloudy or not); bed material (sand, organic, mixed, or mud); water movement (moving or not); water body seasonality (temporal or permanent); depth; alkalinity; conductivity; hardness; light intensity; pH; salinity; and temperature. Nitrate and nitrite levels were not included because of the very low frequency of non-zero measurements (0.7% and 0.2%, respectively).

Censuses, including the GIS coordinates of each inhabited house, were completed in each village in May 2015 (SEM), November 2015 (LUP), or November/December 2016 (all other villages). Using these data, the distance from each water body to the nearest inhabited house, and the number of people living within 50, 100, 250, and 500 m radius buffers from each body were calculated using the R packages sp^40^ and rgeos.^41^ Additionally, presence/absence variables for whether any people lived within each buffer were calculated. As it was not known which, if any, population density measurement at which radius could affect *Ny. nuneztovari* s.l. presence, bivariate models were built for each value at each radius, and the variable with the highest bivariate log likelihood was selected. There was a small amount of missing data in the analysis dataset (2% total, 8% or less for each variable). To account for missing data, multiple imputation was completed using the R package mice^42^ (five imputations, 20 iterations each).

Logistic mixed-effects models were constructed using the imputed datasets with the R package mitml,^43^ using the presence of *Ny. nuneztovari* s.l. as the outcome, and the water body ID as the random intercept to account for multiple collections at the same water body. Bivariate models were used to assess the relationship between each variable and the presence of *Ny. nuneztovari* s.l. The multivariate model was built using a forward stepwise procedure, adding each variable with bivariate p < 0.2 in order of its bivariate log likelihood and retaining variables if p < 0.1 in the final model.

*COI sequence analysis and haplotype network* - *COI* sequences of adult and larval *Ny. nuneztovari* s.l. from the current study (n = 315 of 706 specimens) were edited and checked for stop codons and pseudogenes in Geneious v9.1.4.^44^ Additional *COI* sequences were retrieved from GenBank [Supplementary data (Table V)] for *Ny. dunhami* (n = 12), *Ny. goeldii* (n = 6), and *Ny. nuneztovari* s.l. (n = 43). All sequences were aligned with MUSCLE^45^ using default settings and trimmed in MEGA7 v7.0.26^46^ to the length of the shortest sequence (446 bp). PopART v1.7^47^ was used to construct a median-joining haplotype network,^48^ with epsilon set to 0. Unique sequences for each haplotype containing new sequences from this study have been deposited in GenBank under accession numbers MH723612 to MH723701.

*Ethics* - This study was approved by the Human Subjects Protection Program of the University of California San Diego, La Jolla, and by the Ethical Boards of Universidad Peruana Cayetano Heredia and Asociación Benéfica PRISMA, Lima, Peru.

## RESULTS

*Adult mosquito collections and Plasmodium infection: 2011-2012* - A total of 587 *Ny. nuneztovari* s.l. adults were collected from LUP in 2011-2012 (Table II). Of these, 273 (47%) were confirmed as *Ny. nuneztovari* s.l. by ITS2 PCR-RFLP. The remaining 314 (53%) were not able to be molecularly identified because the specimen was lost in processing or the ITS2 PCR product did not amplify. Additionally, 27 specimens identified morphologically as non-*Ny. nuneztovari* s.l. were determined to be *Ny. nuneztovari* s.l. by ITS2 PCR-RFLP. The initial morphological identification of specimens as *Ny. nuneztovari* s.l. was robust: 246 (95%) of 259 morphologically identified *Ny. nuneztovari* s.l. that could be identified by ITS2 PCR-RFLP were confirmed to be *Ny. nuneztovari* s.l. The remaining 13 specimens were identified as *Ny. darlingi* (n = 6), *Ny. benarrochi* B (n = 3), or *Ny. oswaldoi* s.l. (n = 3); or were unable to be identified (n = 1).

Nearly all collected *Ny. nuneztovari* s.l. adults (579, 99%) were captured outside by HLC or Shannon trap. Since we conducted a mixture of 4- and 12-hour collections, a comprehensive analysis of biting time could not be completed. However, during sampling in February and April 2011, 175 (82%) of 214 *Ny. nuneztovari* s.l. collected by outdoor HLC, and 225 (64%) of 349 by outdoor Shannon trap, were captured between 6-8 pm [Supplementary data (Table IV)].

Of the 273 mosquitoes identified by ITS2 PCR-RFLP as *Ny. nuneztovari* s.l., 270 (99%) were tested for *Plasmodium*. Four specimens, all collected on February 24, 2011 from 6-7 pm by outdoor HLC, were infected, three with *P. falciparum* and one with *P. vivax* [Table II, Supplementary data (Table IV)]. A total of 13 *Ny. nuneztovari* s.l. tested for *Plasmodium* were collected from 6-7 pm on this night (infection rate = 0.31).

*Larval and adult mosquito collections: 2016-2017* - A total of 1,737 molecularly identified *Nyssorhynchus*, *Stethomyia*, and *Anopheles* larvae were collected in 2016-2017; of these, 116 (7%) were *Ny. nuneztovari* s.l., collected from 38 water bodies in all eight villages. These water bodies included active fishponds (n = 17), streams/rivers (14), lagoons (2), swamps (2), abandoned fishponds (2) and puddles (1) [Supplementary data (Table III)].

Excluding 84 collection points at which the water body was dry, there were a total of 403 collection points (water bodies sampled at a time point). Of these, 68 (17%) were positive for *Ny. nuneztovari* s.l. Using bivariate logistic mixed-effects regression, the presence of *Ny. nuneztovari* s.l. was positively associated with (p < 0.2) the presence of non-*Ny. nuneztovari* s.l. Anophelinae species, fish, amphibians, and grass; active fishponds vs. natural water bodies or abandoned fishponds; and partial or total shade; and negatively associated with temporal vs. permanent water bodies and the presence of people in a 250 m radius. In addition, the lowest odds of *Ny. nuneztovari* s.l. presence was in the January-March quarter (the beginning of the rainy season), particularly in 2017. In the multivariate model, the only variables associated with the presence of *Ny. nuneztovari* s.l. (p < 0.1) were quarter of the year, the presence of non-*Ny. nuneztovari* s.l. larvae and amphibians, and the absence of people living in a 250 m radius (Table III).

Only three adult mosquitoes collected in 2016-2017 were identified as *Ny. nuneztovari* s.l. by ITS2 PCR-RFLP: one by outdoor HLC in LIB in March 2016, and two by barrier screen in URC in June 2016. These adults were not tested for *Plasmodium* infection.

*COI sequence analysis and haplotype network* - *COI* barcode region sequences were obtained for 315 (80%) of 392 adults and larvae identified by ITS2 PCR-RFLP as *Ny. nuneztovari* s.l. After discarding 13 low-quality sequences, 363 sequences were retained in the final analysis (from 188 adults collected in 2011-2012, including all four *Plasmodium*-infected adults; all three adults collected in 2016; and 111 larvae collected in 2016-2017). There were 123 haplotypes: 85 included only sequences from this study, five included sequences both from this study and GenBank sequences identified as *Ny. dunhami*, five included only GenBank sequences identified as *Ny. dunhami*, and 28 included a combination of GenBank sequences identified as *Ny. nuneztovari* s.l. and *Ny. goeldii* [Supplementary data (Table V)]. The haplotypes including samples from the present study and *Ny. dunhami* GenBank sequences grouped separately from those including *Ny. nuneztovari* s.l. and *Ny. goeldii* GenBank sequences [Supplementary data (Figure)], indicating that the samples from the present study were all *Ny. dunhami*.

## DISCUSSION

This study provides further confirmation of the presence of *Ny. dunhami* in Peru, and extends its distribution into seven villages in the peri-Iquitos area in addition to LUP.^21^ Though we cannot rule out the presence of other *Ny. nuneztovari* s.l. species in this area, all *Ny. nuneztovari* s.l. mosquitoes that we identified by *COI* sequencing from these eight villages (191 adults and 111 larvae) were *Ny. dunhami*. As morphological keys are unable to differentiate between *Ny. dunhami* and other *Ny. nuneztovari* s.l. species, it is possible that other reports of *Ny. nuneztovari* from Loreto^25^ ^,^ ^27^ ^,^ ^28^ were also *Ny. dunhami*. Additionally, the morphologically similar *Ny. trinkae* ^20^ has been reported infected with *Plasmodium* in the Junín and Madre de Dios Departments of Peru;^25^ ^,^ ^29^ molecular identification methods could be used to determine whether some or all of these specimens, if available, were instead *Ny. dunhami*.

**TABLE II:** Adult *Nyssorhynchus nuneztovari* s.l. collected in Lupuna (LUP), 2011-2012

Month	Collection method	Total nº of *Ny. nuneztovari* s.l. collected (nº confirmed as *Ny. nuneztovari* s.l. by ITS2 PCR-RFLP)	Nº of *Ny. nuneztovari* s.l. confirmed as *Ny. dunhami* by *COI* sequence analysis
February 2011	Human landing catch: inside	1 (0)	0
	Human landing catch: outside	51 (21)	20^*^
	Shannon trap: outside	147 (30)	27
April 2011	CDC light trap: inside	5 (4)	4
	Human landing catch: outside	163 (87)	60
	Shannon trap: outside	202 (130)	77
June 2011	Human landing catch: outside	2 (0)	0
October 2011	Human landing catch: inside	1 (0)	0
	Human landing catch: outside	4 (0)	0
	Shannon trap: outside	1 (0)	0
December 2011	Human landing catch: outside	4 (0)	0
	Shannon trap: outside	1 (0)	0
February 2012	Human landing catch: inside	1 (0)	0
	Human landing catch: outside	3 (0)	0
June 2012	Human landing catch: outside	1 (1)	0
Total		587 (273)	188

*: three infected with *Plasmodium falciparum*, one infected with *P. vivax*; COI: cytochrome c oxidase subunit I; PCR-RFLP: polymerase chain reaction-restriction fragment length polymorphism.

This study is the first report of natural infection of molecularly identified *Ny. dunhami* with *Plasmodium*. The four infected mosquitoes were all collected from the same village during the same hour of one night. It is possible that the infection of these *Ny. dunhami* with *Plasmodium* was an unusual event precipitated by the high densities of *Ny. dunhami* present in LUP in February and April 2011. These densities corresponded with an unusually high infection rate (5.88%) of *Ny. darlingi* in February 2011 in LUP, and an unusually high human biting rate (757 bites per person per night) of *Ny. darlingi* in April 2011 in LUP, despite relatively low numbers of malaria cases in the village in these months.^21^ Although we have continued longitudinal sampling of adult Anophelinae mosquitoes by multiple methods in several villages in peri-Iquitos, including LUP, since 2011,^49^ ^,^ ^50^ we have not collected large numbers of *Ny. dunhami* in any village since April 2011. It is possible that some unknown environmental condition conducive for *Ny. dunhami* reproduction or survival in LUP occurred in 2011. However, our identification of low densities of *Ny. dunhami* larvae and adults in eight villages in peri-Iquitos in 2016-2017 indicates that this species is established in the region, and its potential to be naturally infected with both *P. vivax* and *P. falciparum* suggests that it may be acting as a local or regional secondary malaria vector in peri-Iquitos.

This study was not designed to comprehensively investigate the biting behavior of *Ny. dunhami*. However, our ability to collect this species by HLC and its infection with human *Plasmodium* species confirms that it does blood-feed on humans. In addition, the majority of *Ny. nuneztovari* s.l. in this study were collected in the peridomestic environment from 6-8 pm. This exophagic and early evening biting behavior is consistent with results of other studies of *Ny. nuneztovari* s.l. species in the Amazon,^22^ ^,^ ^25^ ^,^ ^51^ ^,^ ^52^ and might indicate that *Ny. nuneztovari* s.l. species are contributing to residual malaria transmission in peri-Iquitos.

The larval habitat of *Ny. dunhami* has not been previously described. We collected *Ny. nuneztovari* s.l. larvae (over 95% of which were confirmed to be *Ny. dunhami*) from both artificial and natural water bodies in both the rainy and dry seasons. This diversity of larval habitats is consistent with reports of *Ny. nuneztovari* s.l.,^32^ and of *Ny. nuneztovari* s.s. in Colombia and Venezuela.^6^ ^,^ ^8^ ^,^ ^53^. In the current study, *Ny. dunhami* larval habitats were positively associated with the presence of amphibians and other Anophelinae species, and negatively associated with the presence of human dwellings within 250 m. This negative association with humans could be explained by previous reports that *Ny. dunhami* is primarily zoophagic.^22^ ^,^ ^51^ Alternatively, water bodies located far from houses could be more suitable for *Ny. dunhami* oviposition or larval survival due to environmental characteristics not measured during this study or not significantly different in *Ny. dunhami* negative and positive sites due to the relatively small number of positive water bodies. Future research characterizing *Ny. dunhami* habitats and host preference could distinguish between these possibilities.

**TABLE III:** Logistic mixed-effects regression for the presence of *Nyssorhynchus nuneztovari* s.l. larvae in eight localities in peri-Iquitos, 2016-2017

Variable*	Bivariate OR (95% CI)	Bivariate p-value	Multivariate OR (95% CI)	Multivariate p-value
Intercept	-	-	0.01 (0.002, 0.04)	< 0.001
Non-*Ny. nuneztovari* s.l. Anophelinae species present	16.76 (6.92, 40.62)	< 0.001	19.94 (8.00, 49.68)	< 0.001
Active fishpond (ref = natural water body or abandoned fishpond)	4.68 (2.08, 10.53)	< 0.001	-	-
Fish present	3.65 (1.11, 12.02)	0.039	-	-
Amphibians present	2.58 (1.32, 5.03)	0.006	2.27 (1.20, 4.29)	0.012
Quarter (ref = January-March 2017: rainy season)				
January-March 2016 (rainy season)	3.15 (0.98, 10.19)	0.055	3.42 (1.06, 11.05)	0.040
April-June 2016 (rainy season)	4.70 (1.49, 14.85)	0.008	7.65 (2.34, 25.00)	0.001
July-September 2016 (dry season)	4.16 (1.28, 13.51)	0.018	6.15 (1.85, 20.44)	0.003
October-December 2016 (dry season)	4.12 (1.33, 12.75)	0.014	4.45 (1.43, 13.82)	0.010
Grass present	2.77 (1.13, 6.79)	0.026	-	-
Any people living in a 250 m radius	0.42 (0.18, 0.99)	0.048	0.33 (0.16, 0.67)	0.002
Temporal water body (ref = permanent)	0.49 (0.22, 1.12)	0.090	-	-
Shade level (ref = none)				
Partial shade	2.11 (0.76, 5.83)	0.150	-	-
Total shade	2.51 (0.83, 7.56)	0.102	-	-

*: variables not associated with the presence of *Ny. nuneztovari* s.l. (bivariate logistic mixed-effects regression p > 0.2): alkalinity; bed material; cloud cover; conductivity; depth; hardness; light intensity; locality; pH; salinity; temperature; presence of algae, bushes, emergent vegetation, floating vegetation, trees, water movement. CI: confidence interval; OR: odds ratio.

The results of this study highlight the necessity of combining molecular identification techniques with morphological identification when studying the ecology of species in the Nuneztovari Complex. As many countries in South America work towards malaria elimination, it will be essential to increase knowledge of potential local and regional secondary vectors, including *Ny. dunhami*, a task that will require both continued testing of multiple species for *Plasmodium* infection and accurate identification of the mosquito species involved in malaria transmission.
