# Supplementary material for: Nyssorhynchus dunhami: bionomics and natural infection by Plasmodium falciparum and P. vivax in the Peruvian Amazon
Source: Mem Inst Oswaldo Cruz. 2018 Dec 3;113(12):e180380. doi: 10.1590/0074-02760180380 (PMC6276023; doi:10.1590/0074-02760180380)
Supplement: Supplementary file 1 [file 1678-8060-mioc-113-12-e180380-s.pdf]

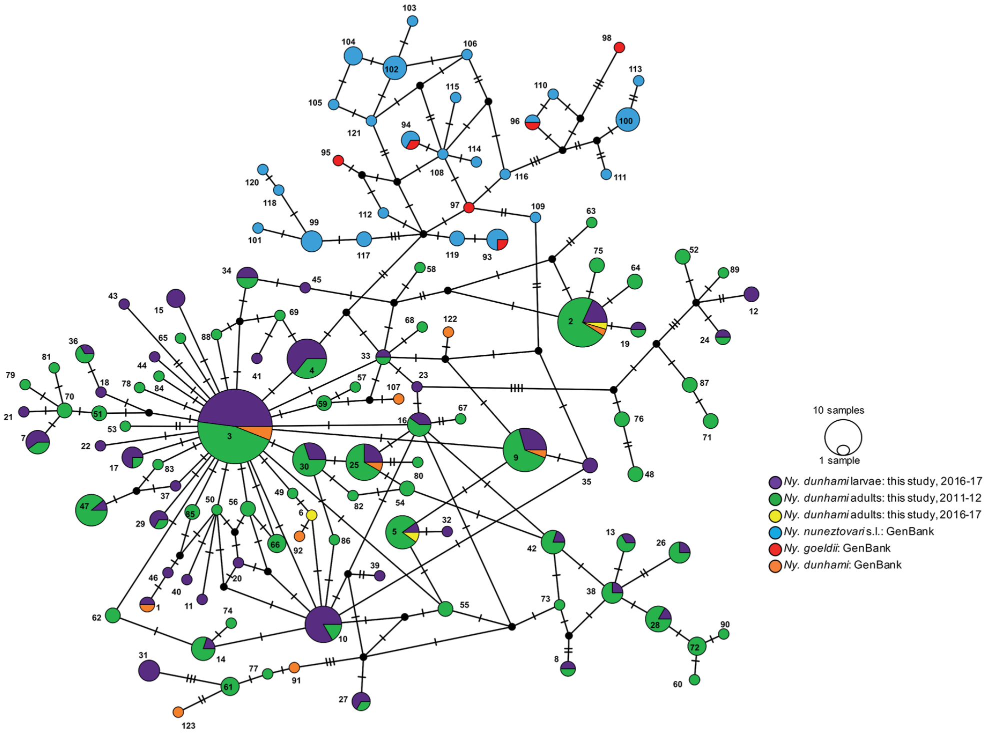

Median-joining COI haplotype network for members of the *Nyssorhynchus nuneztovari* s.l. complex from the current study and from GenBank [n = 363, Supplementary data (Table V)]. Circles represent unique haplotypes and are colored based on sample origin, with the size of the circle proportional to the number of individual sequences sharing the haplotype. Black nodes indicate theoretical missing haplotypes and hash marks represent mutation steps between haplotypes.

TABLE I

All reported collection locations of *Nyssorhynchus dunhami*, with coordinates and references

Locality	Department/State, Country	Latitude	Longitude	Reference
Tefé	Amazonas, Brazil	-3.3207	-64.7235	Type locality. Causey,[15] Lounibos et al.,[17] Ruiz et al.,[20] Scarpassa and Conn[18]
Tabatinga	Amazonas, Brazil	-4.216667	-69.916667	Lounibos et al.[17]
Coari	Amazonas, Brazil	-4.084167	-63.1425	Trindade and Scarpassa,[11] Scarpassa and Conn[18]
Plácido de Castro	Acre, Brazil	-10.333333	-67.183333	Marrelli et al.[19]
Parintins	Amazonas, Brazil	-2.635056	-56.641556	Calado et al.,[5] Foster et al.[3]
Leticia	Amazonas, Colombia	-4.1158	-69.9522	Ruiz et al.[20]
Itaituba	Pará, Brazil	-4.250000	-55.96111	Scarpassa and Conn[18]
Manaus	Amazonas, Brazil	-3.09333	-59.93083	Scarpassa and Conn,[18] Scarpassa et al.[2]
São Miguel	Rondônia, Brazil	-8.60000	-63.81667	Scarpassa and Conn[18]
Lupuna (LUP)	Loreto, Peru	-3.743181	-73.326919	Moreno et al.,[21] This study
Autazes	Amazonas, Brazil	-3.683333	-59.116667	Scarpassa et al.[2]
Cruzeiro do Sul	Acre, Brazil	-7.631440	-72.689688	Maria Anice Mureb Sallum personal communication. Captured April 2015.
Lábrea	Amazonas, Brazil	-7.382054	-64.663073	Maria Anice Mureb Sallum personal communication. Captured August 2015.
Libertad (LIB)	Loreto, Peru	-3.495439	-73.234303	This study
Nuevo Horizonte (NHO)	Loreto, Peru	-4.065089	-73.441292	This study
Salvador (SAL)	Loreto, Peru	-3.438861	-73.15805	This study
Santa Emilia (SEM)	Loreto, Peru	-4.1955833	-74.20955	This study
El Triunfo (TRI)	Loreto, Peru	-4.154186	-73.471739	This study
Urco Miraño (URC)	Loreto, Peru	-3.359542	-73.062008	This study
Visto Bueno (VIB)	Loreto, Peru	-3.448872	-73.316908	This study

CC BY

TABLE II

Adult mosquito collection methods, Lupuna 2011-2012

Month	Night	Collection methods*	Nº of hours of collection**	Total HLC outside collection hours	Total HLC inside collection hours	Total SHA collection hours	Total CDC-LT collection hours
February 2011	1	HLC*** inside	12	20	12	20	0
	2	HLC outside, SHA	4				
	3	HLC outside, SHA	12				
	4	HLC outside, SHA	4				
April 2011	1	HLC outside, SHA, CDC-LT	12	32	0	32	24
	2	HLC outside, SHA	4				
	3	HLC outside, SHA, CDC-LT	12				
	4	HLC outside, SHA	4				
June 2011	1	HLC outside, SHA	12	32	4	28	0
	2	HLC outside & inside	4				
	3	HLC outside, SHA	12				
	4	HLC outside, SHA	4				
August 2011	1	HLC outside, SHA	12	32	0	32	0
	2	HLC outside, SHA	4				
	3	HLC outside, SHA	12				
	4	HLC outside, SHA	4				
October 2011	1	HLC outside, SHA	12	32	4	28	0
	2	HLC outside, SHA	4				
	3	HLC outside, SHA	12				
	4	HLC outside & inside	4				
December 2011	1	HLC outside, SHA	12	32	4	32	0
	2	HLC outside & inside, SHA	4				
	3	HLC outside, SHA	12				
	4	HLC outside, SHA	4				
February 2012	1	HLC outside & inside	12	32	20	16	0
	2	HLC outside & inside	4				
	3	HLC outside, SHA	12				
	4	HLC outside & inside, SHA	4				
April 2012	1	HLC outside, SHA	12	32	10	22	0
	2	HLC outside & inside	4				
	3	HLC outside & inside, SHA	12 (HLC out)/ 6 (HLC in & SHA)				
	4	HLC outside, SHA	4				
June 2012	1	HLC, SHA	12	32	4	32	0
	2	HLC outside & inside, SHA	4				
	3	HLC outside, SHA	12				
	4	HLC outside, SHA	4				
August 2012	1	HLC outside, SHA	4	32	0	32	0
	2	HLC outside, SHA	12				
	3	HLC outside, SHA	4				
	4	HLC outside, SHA	12				
September 2012	1	HLC outside, SHA	12	32	0	32	0
	2	HLC outside, SHA	4				
	3	HLC outside, SHA	12				
	4	HLC outside, SHA	4				
November 2012	1	HLC outside, SHA	12	32	0	32	0
	2	HLC outside, SHA	4				
	3	HLC outside, SHA	12				
	4	HLC outside, SHA	4				
Total	48	-	-	372	58	338	24

*HLC: human landing catch; SHA: shannon trap (outside); CDC-LT: CDC light trap (inside); **12-hour collections: 6 pm - 6 am; 4-hour collections: 6-10 pm; ***HLC collections: 1 collector per hour (if HLC inside & outside were concurrently, 1 collector each inside and outside), with collectors rotating every 2 hours.

TABLE III

Types of water bodies in eight peri-Iquitos localities sampled for *Nyssorhynchus nuenztovari* s.l. larvae, 2016-2017

	Village: nº water bodies sampled (nº positive for *Ny. nuneztovari* s.l. at least 1x)								
Water body type	LIB	LUP	NHO	SAL	SEM	TRI	URC	VIB	Total
Abandoned fishpond*	0	0	1 (0)	1 (1)	0	3 (0)	0	0	5 (1)
Active fishpond*	0	1 (0)	7 (7)	6 (5)	0	2 (2)	1 (0)	0	17 (14)
Active → abandoned fishpond**	0	1 (0)	2 (2)	0	0	1 (1)	1 (1)	0	5 (4)
Lagoon	0	0	0	0	1 (1)	4 (1)	0	0	5 (2)
Puddle	0	3 (1)	2 (0)	0	1 (0)	4 (0)	0	0	10 (1)
Stream/river	8 (4)	15 (6)	1 (0)	2 (1)	4 (0)	1 (0)	6 (2)	4 (1)	41 (14)
Swamp	0	4 (2)	0	0	0	0	1 (0)	0	5 (2)
Total	8 (4)	24 (9)	13 (9)	9 (7)	6 (1)	15 (4)	9 (3)	4 (1)	88 (38)

*: abandoned/active for duration of study; **: active at first sampling, but abandoned over the course of the study. LIB: Libertad; LUP: Lupuna; NHO: Nuevo Horizonte; SAL: Salvador; SEM: Santa Emilia; TRI: El Triunfo; URC: Urco Miraño; VIB: Visto Bueno.
